# Deciphering the alteration of MAP2 interactome caused by a schizophrenia-associated phosphorylation

**DOI:** 10.1016/j.nbd.2024.106731

**Published:** 2024-11-10

**Authors:** Jiali Lyu, Matthew L MacDonald, Shelby Ruiz, Shinnyi Chou, Jordan Gilardi, Serena C Buchwald, Melanie J Grubisha, Robert A Sweet

**Affiliations:** a School of Medicine, Tsinghua University, China; b Translational Neuroscience Program, Department of Psychiatry, University of Pittsburgh, United States of America; c Health Sciences Mass Spectrometry Core, University of Pittsburgh, United States of America; d Department of Neurobiology, University of Pittsburgh, United States of America; e Department of Neurology, University of Pittsburgh, United States of America

**Keywords:** MAP2, Proteomics, Protein-protein interaction, Phosphorylation

## Abstract

Microtubule-associated protein 2 (MAP2) is a crucial regulator of dendritic structure and neuronal function, orchestrating diverse protein interactions within the microtubule network. We have shown MAP2 is hyper-phosphorylated at serine 1782 (S1782) in schizophrenia and phosphomimetic mutation of S1782 in mice (MAP2^S1782E^) is sufficient to impair dendritic architecture. We sought to determine how this hyper-phosphorylation affects the MAP2 interactome to provide insights into the disorder’s mechanisms. We investigated the MAP2 interactome using co-immunoprecipitation and mass spectrometry in MAP2^S1782E^ and MAP2^WT^ mice. We found that S1782E MAP2 led to a substantial disruption of protein-protein interactions relative to WT MAP2. Reduced interactions with PDZ domain-containing proteins, calmodulin-binding proteins, ribosome proteins, and kinesin proteins may all contribute to dendritic impairments induced by S1782E, and may be linked to schizophrenia pathogenesis. Interestingly, novel gain-of-function interactions with PPM1L and KLHL8 nominated these as regulators of phosphoS1782 MAP2 abundance and potential therapeutic targets in schizophrenia.

## 1. Introduction

Microtubule-associated protein 2 (MAP2) is a key regulator of dendritic structure and function in neurons and can serve as a signaling hub by anchoring multiple proteins to the microtubule network (Dehmelt and Halpain, 2004; Morales and Fifkova, 1989; DeGiosio et al., 2022). MAP2 exerts its functions through interactions with various proteins that modulate dendritic function and structure. Primarily, MAP2 stabilizes and promotes the polymerization of microtubules by binding to them (Dehmelt and Halpain, 2004). Additionally, MAP2 can interact with F-actin and with proteins involved in processes crucial to neuronal development such as protein synthesis, cargo transportation, and signal transduction (Lim and Halpain, 2000; Roger et al., 2004; Gumy et al., 2017; Liu et al., 2019).

Phosphorylation plays a pivotal role in regulating MAP2 functions in a manner that is highly phosphosite-specific (Dehmelt and Halpain, 2004; DeGiosio et al., 2022; Sanchez et al., 2000). For example, the primary MAP2 function is binding to microtubules (MT), which promotes MT polymerization and stability (Sanchez et al., 2000). Although changes in overall abundance of MAP2 phosphorylation may impact its MT-binding (Brugg and Matus, 1991), the effects are dependent on the individual phosphosites involved, which may increase, reduce, partially affect, or have no impact on MT regulation (DeGiosio et al., 2023; Komulainen et al., 2014). Phosphorylation also alters MAP2’s interactions with other binding partners. MAP2 interacts with the related microtubule associated protein tau, preventing formation of pathologic tau aggregates in a manner that was inhibited by protein kinase A (PKA) phosphorylation of MAP2 (Mitra et al., 2015; Sarkar et al., 2004). After phosphorylation by MAPK1 (aka ERK2), MAP2’s binding affinity to Fyn, Src, and Grb2 decreased, whereas phosphorylation of MAP2 by PKA had no effect on its binding to Src and Grb2 (Lim and Halpain, 2000; Liu et al., 2019; Zamora-Leon et al., 2001).

We have previously reported that MAP2 is hyperphosphorylated, with phosphorylation at serine 1782 (S1782) being most elevated, in schizophrenia (Grubisha et al., 2021). Phosphomimetic mutation of S1782 (MAP2^S1782E^) in mice induced reduced neuron dendritic length and spine density, both of which are pathologic features present in the cerebral cortex in schizophrenia (Grubisha et al., 2021). We have shown, for example, that S1782E (S426E in the shorter MAP2 isoform, MAP2c) reduces MAP2 MT and actin filament binding, and impairs MT polymerization (DeGiosio et al., 2023; Grubisha et al., 2021). Beyond these altered interactions with the cytoskeleton, MAP2^S1782E^ might further induce these schizophrenia-related pathological changes by altering MAP2 interactions with other proteins involved in regulating dendritic function and structure. To gain deeper insights into the impacts of pS1782, we conducted MAP2 Co-IP followed by liquid chromatography-mass spectrometry assay of cerebral cortex from MAP2^S1782E^ and MAP2^WT^ mice. Selected interactors were validated by Co-IP in HEK293 cells.

## 2. Results

Identification of the interactors of MAP2^WT^ and MAP2^S1782E^ in mouse cerebral cortex.

**Fig. 1.**
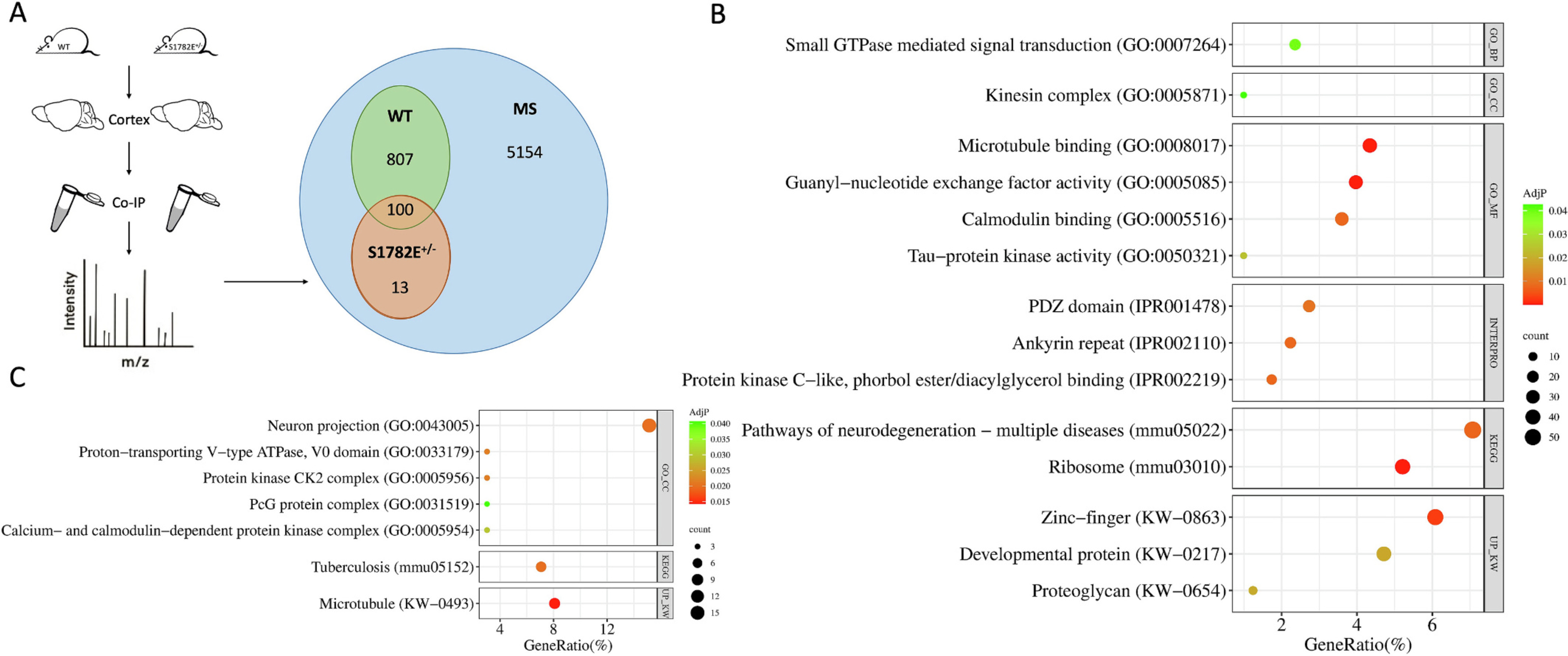
Identification of interactors of MAP2^WT^ and MAP2^S1782E^ in mouse cerebral cortex by CoIP-MS and functional annotation analysis of interactors. (a) Schematic diagram of CoIP-MS procedure. We identified 807 proteins interacting with MAP2^WT^ only, 13 proteins interacting with MAP2^S1782E^ only, and 100 proteins interacting with both. (b) Dot plot showing GO terms, KEGG pathways, INTEPRO terms, and UP terms enriched in 807 MAP2^WT^ unique interactors. (c) Dot plot showing GO terms, KEGG pathways, INTEPRO terms, and UP terms enriched in 101 MAP2^WT^ and MAP2^S1782E^ common interactors.

Phosphorylation of MAP2 is known to alter its structure, function, and immunoreactivity (Sanchez et al., 2000; Grubisha et al., 2021; Ramkumar et al., 2018). We have previously shown that MAP2 is hyperphosphorylated in schizophrenia (SZ), with phosphorylation at serine 1782 (S1782) being increased ~7-fold in schizophrenia subjects compared to non-psychiatric controls (Grubisha et al., 2021). In the current study, we comprehensively investigated how phosphomimicry at S1782 alters the MAP2 interactome. We used co-immunoprecipitation coupled with mass spectrometry (CoIP-MS) experiments to identify MAP2 binding partners in both S1782E^+/−^ MAP2 (Grubisha et al., 2021) and wild-type (WT) MAP2 mice (*N* = 8 mice per group). As a control, we performed CoIP using beads without MAP2 antibody (N = 8 mice total, 4 per genotype). We utilized SAINTq (Choi et al., 2011; Teo et al., 2016) to calculate the posterior probability (PP) of protein-protein interactions compared to no-antibody control. We identified 100 candidate proteins interacting with both MAP2^WT^ and MAP2^S1782E^ (common interactors), 807 interacting with MAP2^WT^ only, and 13 proteins interacting with MAP2^S1782E^ only (Fig. 1A and Table S1). This suggests that S1782E acts primarily as a loss of function modification, with only a much smaller quantity of proteins as potential gain of function interactions. Of interest, 53 of the detected MAP2 interactors were annotated as GWAS risk genes for Sz (Table S1). Of the proteins with the greatest reduction in PP between MAP2^WT^ and MAP2^S1782E^ were Gdf7 (Uniprot Accession = P43029), Ccdc33 (Q3ULW6), and Cplane1 (Q8CE72, Saintq PP (BDFR) in S1782E^+/−^ group = 0.266 (0.425), 0.276 (0.420), and 0.291 (0.409), respectively, Saintq PP (BDFR) in WT group = 0.979 (0.006), 0.920 (0.025), and 0.881 (0.039), respectively). Of the proteins with the greatest increase in PP in MAP2^S1782E^ were Tmprss15 (P97435), Nid1 (P10493), and Ppm1l (Q8BHN0) (Saintq PP (BDFR) in WT group = 0.333 (0.279), 0.666 (0.130), and 0.667 (0.128), respectively, Saintq PP (BDFR) in S1782E^+/−^ group = 0.9075 (0.047), 0.999 (<0.001), and 0.945 (0.0136) respectively). (See Table S1.)

We next conducted functional annotation analysis of the set of MAP2^WT^ only interactors to better understand the functions impaired by the loss of MAP2 interactions present in MAP2^S1782E^ (Fig. 1B and Table S2, see also Fig. S1 for a visual representation of the network of interactions among these proteins). Consistent with our prior report (Grubisha et al., 2021), MAP2^WT^ only interactors were enriched for interactions with microtubule binding proteins (GO:0008017 – adjusted *p* = 9.1 × 10^−5^, *N* = 34 proteins). We also newly identified that the enrichments of MAP2^WT^ only interactors for proteins associated with other important functional scaffolds were lost in MAP2^S1782E^. These included proteins associated with the post-synaptic density (PDZ domain containing proteins IPRO: IPRO001478, adjusted *p* = 0.0096, *N* = 22) and Calmodulin binding proteins (GO:0005516, adjusted *p* = 0.008, *N* = 29). Of note, this loss of function in MAP2^S1782E^ did not extend to several calcium- and calmodulin- dependent protein kinases, which were enriched in the set of interactors common to both MAP2^WT^ and MAP2^S1782E^ (GO:0005954, adjusted *p* = 0.0296, Camk2a (Uniprot: Q9UQM7), Camk2b (Q13554), and Camk2g (Q13555) proteins). Finally, enrichment analysis identified MAP2^WT^ only interactors were enriched for the Kinesin complex (GO:0005871, adjusted *p* = 0.043, *N* = 8) gene set, containing multiple kinesin proteins known to be regulated by MAP2 (Gumy et al., 2017).

Validation of binding of specific identified interactors with human MAP2^WT^ and human MAP2^S1782E^.

We next sought to validate our SAINTq analysis of probable protein-protein interactions. We selected for validation two identified interactors that showed increased selectivity for MAP2^S1782E^ relative to MAP2^WT^ and which, based on their known functions, were likely to be direct interactors with MAP2. These were Ppm1l and Klhl8 (Saintq PP (BDFR) in S1782E^+/−^ group 0.945 (<0.001) and 0.993 (0.001) and in WT group 0.667 (0.128) and 0.8333 (0.055)). Ppm1l (Protein Phosphatase, Mg^2+^/Mn^2+^ Dependent 1 L) is a phosphatase that may potentially target pS1782 MAP2 for dephosphorylation. Klhl8, Kelch Like Family Member 8, is an E3 ubiquitin ligase adaptor that may target S1782E MAP2 for degradation (Schaefer and Rongo, 2006; Nam et al., 2009). Of the proteins with decreased interaction with MAP2^S1782E^ relative to MAP2^WT^, we selected Kif5c (Saintq PP (BDFR) in S1782E^+/−^ group 0.723 (0.165) and in WT group 0.910 (0.029), given evidence that MAP2 normally interacts with members of the kinesin family of motor proteins to regulate their function in dendritic microtubule-based transport (Gumy et al., 2017).

**Fig. 2.**
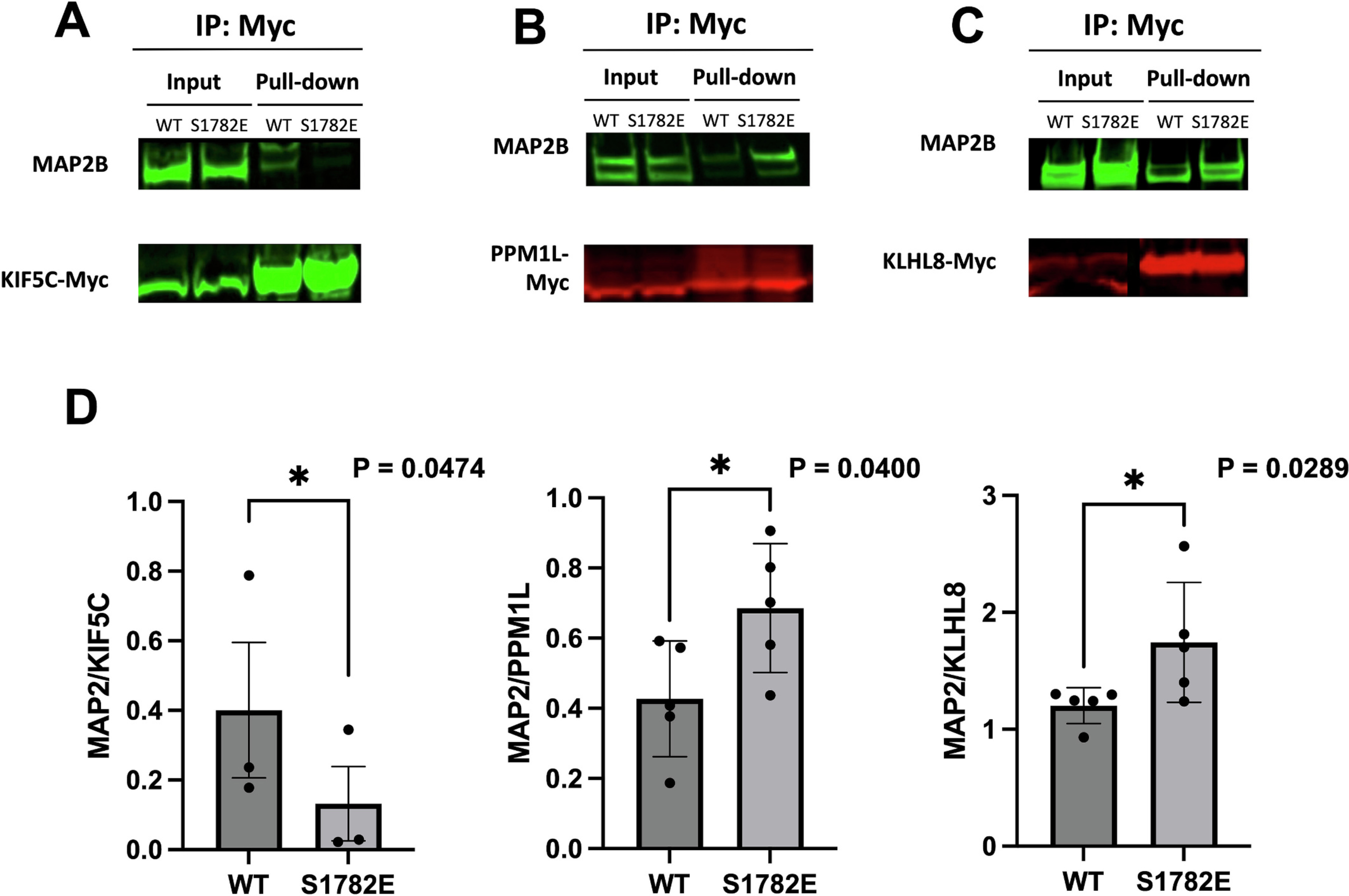
Validation of binding of specific identified interactors (a-c) Representative WB results of KIF5C Co-IP, PPM1L Co-IP, and KLHL8 Co-IP (d) Quantification of MAP2 and bait pull-down in each group, represented as mean ± SD, Student *t-*test *p* < 0.05.

Using HEK cells, we transiently expressed plasmids containing MAP2^WT^ or MAP2^S1782E^ and a myc-tagged potential interactor followed by co-IP. Myc-tagged magnetic beads were used to bind potential interactors, and western blot was performed using antibodies directed against MAP2 and against Myc. Protein interaction was quantified by densitometry of MAP2 (WT or S1782E) compared to the interacting protein. As predicted by our MS results, KLHL8 and PPM1L had significantly higher interactions of MAP2^S1782E^ compared to MAP2^WT^ (Fig. 2B, C, and D, see also Fig. S3 and S4), respectively, while KIF5C exhibited significantly lower interaction of MAP2^S1782E^ (Fig. 2A and D, see also Fig. S2). Taken together, these data support stronger KLHL8 and PPM1L interactions and weaker KIF5C interaction due to the S1782E mutation.

Orthogonal validation of binding of specific identified interactors with MAP2 in human brain.

**Fig. 3:**
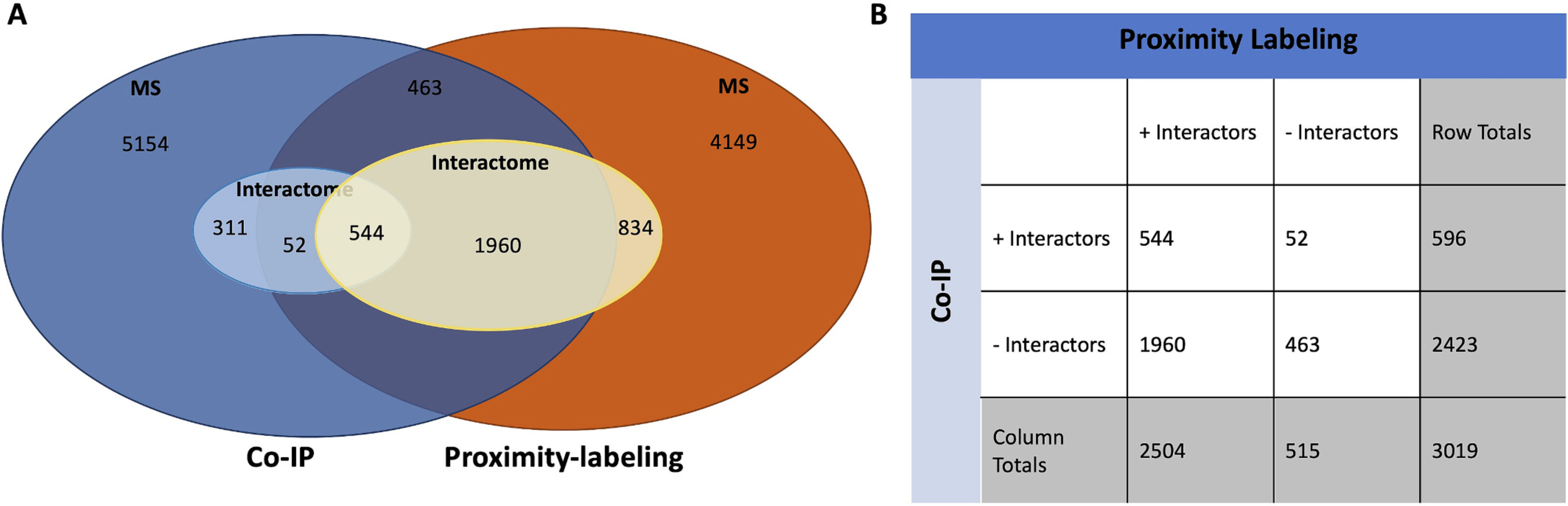
Overlaps of interactors of MAP2WT identified by CoIP-MS and proximity-labeling. (a) The Venn diagram illustrates the overlap of proteins interacting with MAP2^WT^ as identified by two methods: Co-immunoprecipitation (Co-IP) and proximity labeling. A total of 5154 proteins were identified by Co-IP (dark blue circle), while 4149 proteins were identified by proximity labeling (orange circle), 3019 proteins were detected by both methods. We identified 907 proteins interacting with MAP2^WT^ via Co-IP (light blue circle) and 3338 proteins interacting with MAP2^WT^ via proximity-labeling (yellow circle). Of the 3019 proteins identified in common across the two methods, 544 proteins were identified as MAP2^WT^ interactors by both methods. Conversely, 463 of the 3019 proteins identified in common were found to not interact with MAP2^WT^ by both methods (purple shading). (b) Contingency table showing the distribution of MAP2 interactors identified by both Co-IP and Proximity Labeling (χ^2^ = 36.4, df = 1, *p* < 0.00001). (For interpretation of the references to colour in this figure legend, the reader is referred to the web version of this article.)

To ensure the robustness of our findings, we extended our analysis of the MAP2 interactome using an alternate approach, proximity labeling, in cerebral cortex obtained at autopsy from 2 individuals without a known history of psychiatric illness. The tissue samples were labeled for MAP2 using an alternate antibody to MAP2, SMI-52, which we have previously validated as having specificity for MAP2 (McKinney et al., 2019) and effectively labeling it in fixed sections (see also Fig. S5). Samples were biotinylated, reverse fixed, proteins were pulled down using streptavidin beads, and identified via mass spectrometry. We detected 3338 candidate proteins interacting with MAP2 (SAINTq BFDR q < 0.05, Table S3). 544 proteins were common to CoIP-MS and proximity-labeling-MS (Fig. 3), a significant overlap (χ^2^ = 36.4, df = 1, *p* < 0.00001).

Co-IP may not detect weak and/or transient interactions, whereas proximity-labeling may identify noninteracting nearby proteins, potentially explaining the differences between the MAP2 interactomes identified by two approaches. We thus restricted our further analysis to the 544 interactors that were also identified by Co-IP. Functional annotation analysis of the overlapping interactors yielded results highly overlapping with those identified in our mouse co-ip experiment. Specifically we continued to observed enrichment in microtubule binding proteins (GO:0008017 – adjusted *p* = 5.9 × 10^−4^, *N* = 34 proteins), ribosome proteins (GO:0022626, adjusted *p* = 2.8 × 10^−10^, *N* = 35), PDZ domain containing proteins (IPRO: IPRO001478, adjusted p = 5.9 × 10^−04^, *N* = 22), calmodulin-binding proteins (GO:0005516, adjusted *p* = 0.0002, N = 34), and others (see Table S4 for complete list of enriched proteins). The enrichment findings are highly consistent across the interactomes and align with the known functions of MAP2.

## 3. Discussion

While previous studies have identified protein interactors and the protein-interaction domains of MAP2 by Co-IP (Lim and Halpain, 2000; Liu et al., 2019; Grubisha et al., 2021; Zhong et al., 2009; Nielsen et al., 2002), how these interactions may be affected by posttranslational modifications such as site-specific phosphorylations have yet to be fully elucidated. Previous work observed hyperphosphorylation at S1782 in subjects with schizophrenia (Grubisha et al., 2021). While noting that phosphomimicry at S1782 was sufficient to alter MAP2 structure and functions, and induce structural deficits in neurons, how it altered MAP2 interactions to achieve these effects was not known (Grubisha et al., 2021). Our study focused on understanding the effect of hyperphosphorylation at S1782 on the MAP2 interactome using phosphomimicry followed by co-IP and MS in mouse brain expressing either MAP2^WT^ or MAP2^S1782E^. We identified 807 candidate proteins interacting with MAP2^WT^ only, whereas only 100 proteins interacted with both MAP2^WT^ and MAP2^S1782E^ (common interactors), and only 13 proteins interacting with MAP2^S1782E^ only, suggesting an overall loss of function in protein-protein interactions as a result of phosphomimicry at S1782.

Several known MAP2 interactors were identified in our dataset, including ribosome proteins and growth factor receptor-bound protein 2 (Grb2) (Lim and Halpain, 2000; Grubisha et al., 2021), thus increasing confidence in our results. In addition, using a heterologous expression system, we validated proteins from our mouse brain dataset with both increased and decreased interactions with MAP2 ^S1782E^ relative to MAP2^WT^. Consistent with our mouse brain Co-IP experiment, PPM1L and KLHL8 both exhibited increased pulldown by MAP2^S1782E^. We note that a limitation of confirming interactions only via reciprocal in vitro overexpression is an inability to establish that the interactions are present within specific sub-cellular compartments at relevant in vivo concentrations. Another potential limitation to the current report is that some of the lost interactions with MAP2^S1782E^ are due to reduced levels of the interactors, as have previously reported that MAP2^S1782E^ mice have reduced levels of multiple cerebral cortex proteins (Grubisha et al., 2021). However, it is clear from our confirmatory experiment with KIF5C that such an effect does not explain all of the reduced interactions we identified. Further enhancing confidence in our results, an assessment of the MAP2 interactome in fixed human cerebral cortex tissue using an alternate MAP2 antibody and an alternate approach to identifying interactors, proximity labeling, confirmed the presence of a conserved set of MAP2 interactors enriched for proteins with overlapping functions.

S1782E induces a widespread MAP2 loss of interactions, potentially impacting Sz pathogenesis.

**Fig. 4.**
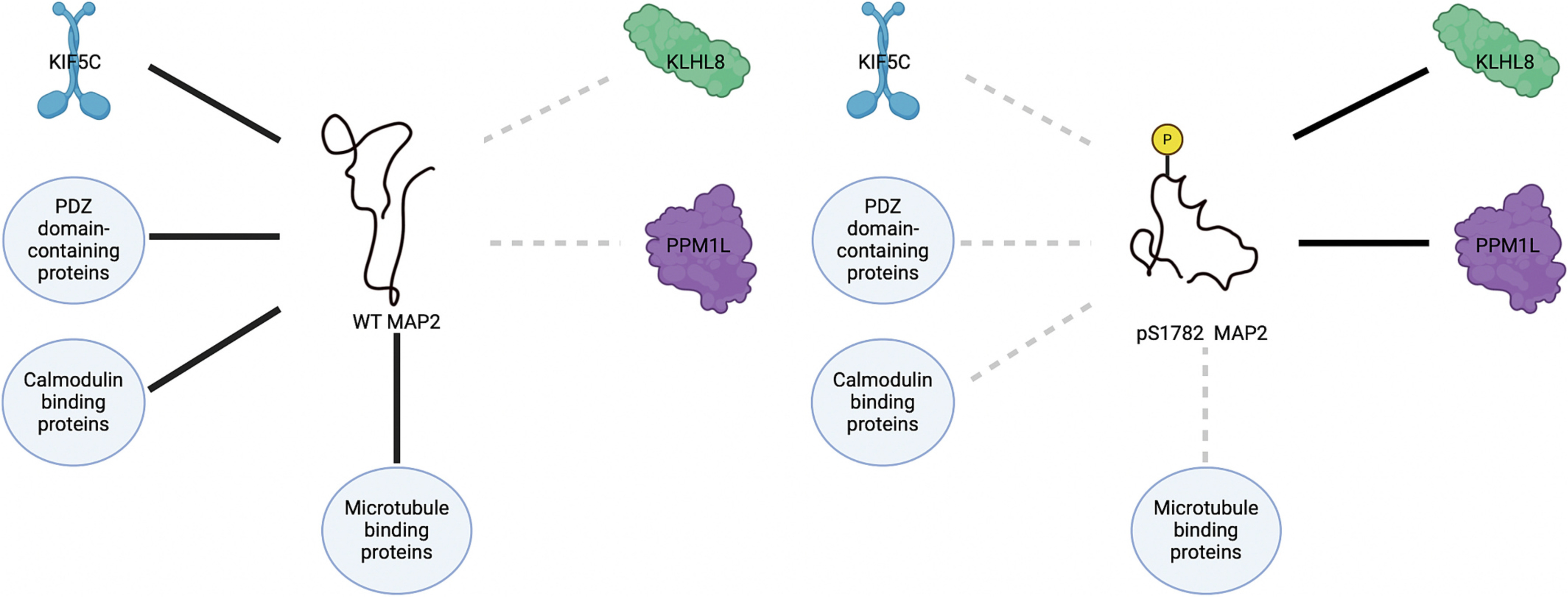
Schematic Summary Figure Disrupted Protein Interactions and Novel Targets of MAP2S1782E in Schizophrenia This figure illustrates the disruption of the MAP2 interactome caused by the phosphomimetic MAP2S1782E mutation. On the left, wild-type MAP2 interacts with PDZ domain-containing proteins, calmodulin-binding proteins, ribosomal proteins, and kinesin proteins (represented by solid black lines). On the right, the S1782E MAP2 mutation alters MAP2’s structure, resulting in the disruption of these protein-protein interactions (dashed grey lines). Additionally, novel gain-of-function interactions are observed between S1782E MAP2 and the proteins PPM1L and KLHL8 (solid black lines).

Taken together, these findings suggest that our prior computational models of changes to MAP2’s structure due to phosphorylation or phosphomimicry at S1782 are sufficient to alter the MAP2 interactome (Fig. 4) and that the excess phosphorylation at S1782 we have observed in Sz is likely to induce loss of core set of MAP2 interactions in that disorder (Grubisha et al., 2021). Among the MAP2 interacting protein clusters for which phosphomimicry at S1782 served as a loss of function (LOF), were PDZ domain-containing proteins, calmodulin-binding proteins, and ribosome proteins.

PDZ domain-containing proteins are known to be important components of the postsynaptic density (PSD), which plays a crucial role in synaptic function and plasticity (Lee and Zheng, 2010; Kim and Sheng, 2004). The PDZ domain serves as a recognition and binding module for specific PDZ-binding motifs, enabling it to assemble as a molecular complex that acts as a scaffold (Kim and Sheng, 2004). Meanwhile, the PDZ domain has the capacity to engage with numerous signaling proteins and membrane receptors, facilitating the organization of post-synaptic signaling processes (Lee and Zheng, 2010; Kim and Sheng, 2004). A loss of binding between MAP2 and PDZ domain-containing proteins, as seen with MAP2^S1782E^, could potentially disrupt the proper distribution of MAP2 within the neuronal cells. For example, during long-term potential (LTP), MAP2 is translocated to postsynaptic region, and this is linked to several LTP-induced consequences, including spine enlargement and surface insertion of AMPARs (Kim et al., 2020). Consequently, the loss of binding to PDZ domain-containing proteins caused by S1782E may exert an influence on LTP.

Relatedly, we found that MAP2^S1782E^ also loses binding to Ank3 (Saintq PP (BDFR) in WT group = 0.9222 (0.0248, Saintq PP (BDFR) in S1782E^+/−^ group = 0.7548 (0.1469)). Ank3 helps to organize the axon initial segments (AIS), which in turn regulates traffic and distribution of proteins in neurons (Huang and Rasband, 2018). The deletion of this protein can cause the loss of the polarity of MAP2 distribution in neurons, leading to MAP2 entering axons (Hedstrom et al., 2008; van Beuningen et al., 2015). Taken together with the reduction in PSD protein binding, this suggests that misdistribution of S1782E MAP2 could occur and contribute to the impairments in synaptic organization and function, processes known to be disrupted in Sz (Javitt and Sweet, 2015).

Calmodulin, similar to PSD 95, functions as a scaffold for protein interactions (Villalobo et al., 2018). Its four-arm structure allows it to serve as a bridge between different calmodulin binding domain containing proteins, facilitating their interaction and coordinated activity (Villalobo et al., 2018). MAP2 has been shown to interact with calmodulin in a calcium-dependent manner (Sobue et al., 1985). Our findings indicate that the S1782E mutation might interfere with the interaction between MAP2 and calmodulin or interfere with the binding of calmodulin to its other interactors, disrupting the interactions and coordination of MAP2 with multiple calmodulin-binding proteins. The set of calmodulin-binding proteins interacting only with MAP2^WT^ (Table S2) include many with established roles in dendritic development and plasticity. Thus, the disruption in the interaction with calmodulin binding proteins due to the S1782E mutation likely contributes to the altered dendritic morphologies present in MAP2^S1782E^ mice (Grubisha et al., 2021).

Finally, our analysis revealed a LOF with S1782E in protein clusters of known MAP2 interactors such as proteins associated with kinesins. This could be caused by loss of direct interactions or via intermediaries. For kinesins, for example, this loss could be achieved by the loss of MAP2^S1782E^ binding to microtubules (DeGiosio et al., 2021).Regardless, the reduced interaction of MAP2^S1782E^ with kinesins indicates that MAP2^S1782E^ may also negatively impact the regulation of cellular transportation (Gumy et al., 2017; Grubisha et al., 2021). Kinesin-mediated transport is vital for delivering AMPA receptors to specific regions of neurons, ensuring proper synaptic function (Brachet et al., 2021; Hirokawa et al., 2010; Hoerndli et al., 2013). There is evidence for failed forward delivery of these receptors to synapses in schizophrenia (Hammond et al., 2010; Zeppillo et al., 2020; MacDonald et al., 2020).

S1782E leads to several gain-of-function interactions, indicating potential novel therapeutic targets for Sz.

Although largely revealed to be a LOF mutation, S1782E was observed to act as a gain-of-function (GOF) for several interactors, most notably PPM1L and KLHL8. To our knowledge, ours is the first report of PPM1L and KLHL8 as interactors of MAP2, and of S1782E acting as a GOF for these interactions. Both PPM1L and KLHL8 serve to regulate protein activity and/or levels. PPM1L is a phosphatase involved in the dephosphorylation of various target proteins (Flajolet et al., 2003), while KLHL8 is the substrate adaptor of CUL3 E3 ubiquitin ligase that participates in protein ubiquitination and degradation, previously shown to mediate the degradation of Rapsyn and AMPARs in neurons (Schaefer and Rongo, 2006; Nam et al., 2009; Canning et al., 2013). The increased binding of S1782E MAP2 to PPM1L and KLHL8 suggests a potential regulatory mechanism for MAP2 dephosphorylation and targeted ubiquitination/degradation, respectively. While no prior studies have identified specific roles of PPM1L and KLHL8 in schizophrenia, our data nominate PPM1L and KLHL8 as potential regulators of MAP2 phosphorylation levels, and thus as novel targets for further exploration to reverse schizophrenia-related hyperphosphorylation of MAP2. Future studies using, for example, isogenic manipulation of human induced pluripotent stem cell derived neurons to investigate the functional consequences of these alterations, would provide a more comprehensive understanding of the role of PPM1L and KLHL8 in the regulation of hyperphosphorylated MAP2 and whether it may reflect a therapeutic strategy to enhance neuronal health in Sz.

## 4. Conclusion

In conclusion, the phosphomimetic S1782E mutation in MAP2 leads to an overall LOF in the MAP2 interactome. Decreased binding to kinesins, PDZ domain-containing proteins, and calmodulin-binding proteins may contribute to the pathogenesis of schizophrenia. Simultaneously, enhanced interactions with KLHL8 and PPM1L offer potential avenues for innovative treatment aimed at reducing the site-specific hyperphosphorylation of MAP2 seen in Sz. Understanding the functional consequences of these alterations can provide valuable insights into the role of MAP2 in schizophrenia and provide new possibilities for therapeutic interventions.

## 5. Methods

### 5.1. Animal cohort and tissue preparation.=

The generation of S1782E^+/−^mouse models was previously described in (Grubisha et al., 2021) (and are available upon request). Animals were identified with metal ear tags and tail snip DNA samples were obtained for genotyping by PCR prior to weaning at approximately postnatal day 21. Genotypes of all mice included in studies were confirmed following sacrifice. Animals were under specific pathogen free conditions and housed in standard microisolator caging (Allentown Caging Equipment, Allentown, NJ, USA) in groups of up to 4 males or 5 females, maintained on a 12-h light/dark cycle, and were provided with food and water ad libitum. All experimental procedures were approved by the Institutional Animal Care and Use Committee at the University of Pittsburgh.

To produce the study cohort, S1782E−/+ animals were crossed with purchased C57/Bl6J mice. This prevents genetic drift and allows use of littermates, as all offspring are of a desired study genotype. 8 mice per genotype (4 M, 4F), were aged to 4 weeks prior to sacrifice by lethal CO_2_ inhalation followed by rapid decapitation. Whole mouse brains were then extracted, hemisectioned, snap frozen and stored at −80 °C until use.

### 5.2. HEK cell cultures and transfections

HEK 293 cell line was purchased from ATCC and maintained in RPMI-1640 supplemented with 10 % fetal bovine serum (ThermoFisher Scientific) without antibiotics. For transfections they were seeded at a density of 2.2 × 10^8^ cells into 10-cm dishes and serum-starved for 18 h before transfection. Lipofectamine 2000 (ThermoFisher Scientific, 11668027) was used for transfection according to the manufacturer’s instructions (60 μL/dish, xx μg DNA/well). The media was replenished 24 h post-transfection. Transfected cell lysates were used to run western blot to determine the transfection efficiency.

### 5.3. DNA constructs

Plasmids used in the experiments are listed in the following table (Table 1):

MAP2B S1782E mutation was generated with QuikChange Lightning Site-directed Mutagenesis Kit (Agilent Technologies). Sequences of all plasmids were confirmed via Sanger sequencing.

### 5.4. Co-IP

#### Mouse brain:

mouse cortex tissues were washed by PBS and homogenized in RNase-treated sample buffer (0.125 M Tris-HCl pH 7, 2 % SDS, protease inhibitor (Sigma, P8340) and phosphatase inhibitor (Sigma, P0044)), with a pre-cooled micropestle. The lysates were passed through a 23G needle for several times to ensure a complete lysis. Brain homogenates were incubated on ice for 10 min and then centrifuged at 14,000 *g* for 20 min at 4 °C. The supernatants were further purified by a second centrifugation at 12,000 *g* for 10 min at 4 °C. The protein concentration of purified supernatants was determined by BCA Assay (Pierce, 23,225). 2 mg of each supernatant protein was diluted in 500 μL of sample buffer and pre-cleared with 1.5 mg of control Dynabeads (ThermoFisher Scientific, 14311D). 125 μL of each pre-cleared sample was incubated with either HM-2 (Abcam ab11267, 8 μg antibody per mg of beads) conjugated or no-antibody control Dynabeads. The co-IP complexes were washed with PBS buffer for 3 times and eluted with SDS sample buffer (without triton-x). The eluate was stored at −80 ֯C until use.

#### HEK 293 cells:

48 h post-transfection, HEK 293 cells were harvested with IP lysis buffer (20 mm Tris-HCl, pH 7.5, 150 mm NaCl, 1 % Triton X-100) supplied with protease inhibitor (Sigma, P8340) and phosphatase inhibitor (Sigma, P0044) and incubated on ice for 20 min. The lysate was centrifuged at 13,000 rpm for 15 min at 4 °C. 50 μL of the supernatant was preserved as the input control in western blotting and the rest of the supernatant was incubated with 75 μL of Myc-antibody magnetic beads (ThermoFisher Scientific, cat#88842). The co-IP complex was washed with IP lysis buffer for 3 times and eluted with SDS sample buffer by boiling for 10 min. The eluate was stored at −80 ֯C until use.

### 5.5. Western blotting

13 μL of eluate from HEK cell co-IP was separated by SDS-PAGE on a 3 to 8 % tris-acetate gel (ThermoFisher Scientific, E03752) and transferred to PVDF membrane (Millipore, IPVH00010). The membrane was blocked with Licor blocking buffer for 1 h at room temperature. Primary antibodies were diluted in ThermoFisher blocking buffer and incubated with membranes on a shaker at 4 °C overnight. The following primary antibodies were used: HM-2 MAP2 antibody (Abcam. Ab11267, 1:1000 dilution), Myc antibody (Santa Cruz, sc-40, 1:4000 dilution). Membranes were washed x5 in TBST and incubated with fluorescent goat-anti-mouse (Licor, 926–32,210) antibody diluted in Licor blocking buffer (1:10000 dilution) for 1 h at room temp. Membranes were imaged on a Licor Odyssey system and band intensity was quantified using Imagelab software.

### 5.6. Human tissue for proximity labeling

Human brain specimen sources were collected during autopsies conducted at the Allegheny County Medical Examiner’s Office, Pittsburgh, PA. Samples from two adult male human subjects (49 and 62 years of age; postmortem interval [PMI] 17.8 and 10.6 h, respectively) were obtained following informed consent for brain donation from the next of kin. Based on the information available from the Medical Examiner and neuropathology exam, neither decedents had a known brain disorder, Following retrieval of brain specimens, the left hemisphere was cut into 1.0–2.0 cm-thick coronal blocks and fixed for 48 h in phosphate-buffered 4 % paraformaldehyde at 4C. Tissue blocks were subsequently immersed in graded cold sucrose solutions then stored at −30C in a cryoprotectant solution until sectioning. Tissue blocks containing the superior temporal gyrus (containing the primary auditory cortex) were sectioned coronally at 40 um on a cryostat, and every 40th section was stained for Nissl substance with thionin to serve as anatomical references. Unstained sections were stored until processed for immunohistochemistry at −30 °C in the same cryoprotectant solution as above.

All methods were carried out in accordance with relevant guidelines and regulations from the University of Pittsburgh’s Committee for the Oversight of Research and Clinical Trials Involving Decedents and Institutional Review Board.

### 5.7. Immunohistochemistry

Three tissue sections per subject were labeled with antibodies and subjected to biotinylation using the Tyramide SuperBoost Kit (Thermo Fisher Scientific, Waltham, MA, #B40911). Sections were washed in 0.1 M phosphate-buffered saline (PBS). Sections were then immersed in 1 % sodium borohydride for 30 min at room temperature (RT) to reduce background autofluorescence, followed by membrane permeabilization with 0.3 % Triton X-100 in PBS for 30 min at RT. To block endogenous peroxidases, sections were incubated with 3 % hydrogen peroxide (H_2_O_2_) for 20 min in the dark. Sections were blocked with 20 % normal goat serum (NGS) and 20 % normal human serum (NHS) in PBS with 1 % bovine serum albumin (BSA), 0.1 % glycine, and 0.1 % lysine for 2.5 h at RT to reduce nonspecific antibody binding, then incubated for 72 h at 4 °C in PBS containing 5 % NGS, 5 % NHS, 1 % BSA, 0.1 % glycine, and 0.1 % lysine and a primary antibody against MAP2 (anti-MAP2-mouse; 1:500, BioLegend, San Diego, CA, #SMI-52), a ubiquitous marker of neurons. Control conditions were not incubated with primary antibodies. We previously demonstrated successful and specific MAP2 labeling in human postmortem studies using these antibodies (McKinney et al., 2019).

Following primary antibody incubation, sections were rinsed 4 × 30 min in PBS and incubated for 24 h at 4 °C in PBS containing 5 % NGS, 5 % NHS, 1 % BSA, 0.1 % glycine, 0.1 % lysine, and goat host secondary antibodies conjugated to poly horseradish peroxidase (HRP) (antimouse, (1×), Thermo #B40961). Sections were again rinsed 4 × 30 min in PBS and biotinylated according to the manufacturer’s instructions of the Tyramide SuperBoost Kit. Briefly, equal volumes of tyramide and H_2_O_2_ stock solutions were mixed with a reaction buffer and allowed to incubate with sections for 5 min. Sections were then incubated briefly with a stop reagent and rinsed 3 × 3 min in PBS. A small piece of each section was trimmed for imaging validation and the remaining tissue was additionally removed of all white matter and stored at −80 °C.

Trimmed imaging sections were incubated for 24 h at 4 °C in PBS containing 5 % NGS, 5 % NHS, 1 % BSA, 0.1 % glycine, 0.1 % lysine, a goat host secondary antibody conjugated to Alexa-568 (1:500; Invitrogen, Grand Island, NY, A-11004), and streptavidin conjugated to Alexa 488 (1:500; Thermo Fisher Scientific, Waltham, MA, #S11223). Trimmed sections were again rinsed 4 × 30 min in PBS, mounted on slides, cover slipped (ProLong Gold antifade reagent, Invitrogen), sealed with clear nail polish along coverslip edges, and stored at 4 °C until imaged.

### 5.8. Confocal microscopy

Microscopy equipment and capturing parameters were as previously described. Data were collected using a 10 × objective mounted on an Olympus BX51Wl upright microscope (Olympus America Inc., Center Valley, PA) equipped with an Olympus spinning disk confocal unit, Hamamatsu Orca R2 camera (Hamamatsu, Bridgewater, NJ), MBF CX9000 front mounted digital camera (MicroBrightField Inc., Natick, MA), BioPrecision2 XYZ motorized stage with linear XYZ encoders (Ludl Electronic Products Ltd., Hawthorne, NY), excitation and emission filter wheels (Ludl Electronic Products Ltd., Hawthorne, NY), Sedat Quad 89,000 filter set (Chroma Technology Corp., Bellows Falls, VT), and a Lumen 220 metal halide lamp (Prior Scientific, Rockland, MA). The equipment was controlled by SlideBook 6.0 (Intelligent Imaging Innovations, Inc., Denver, CO).

### 5.9. Reverse fixation

Tissue sections were weighed and supplemented with twice the volume (100 mg tissue to 200 uL buffer) of 1 % lysis buffer (1 % sodium dodecyl sulfate (SDS), 125 mM triethylammonium bicarbonate (TEAB), 75 mM NaCl, and protease and phosphatase inhibitors (Sigma, Protease inhibitor (0.2 %) #I3786; Sigma, Phosphatase inhibitor 2 (0.5 %) #P5726; Phosphatase inhibitor 3 (0.5 %) #P0044) and equal amounts of stainless-steel beads (Next Advance, 0.2 mm beads, #SSB02). Tissue was then homogenized with the bullet blender (Next Advance, #BT24M) at a speed of 12 for 5 min followed by a speed of 10 for 3 min. Samples were then probe sonicated three times, heated for 1 h at 90oC, and cleared by centrifugation at 13,000 *g* for 10 min at RT. Supernatant was transferred to a fresh tube and diluted to a final concentration of 0.5 % lysis buffer.

### 5.10. Streptavidin enrichment

Two technical replicates per labeled tissue section were subjected to enrichment and all further sample processing steps. Following a microBCA protein assay, 300 μg of lysate was aliquoted into 250 uL of 1 % lysis buffer. 150 uL of streptavidin magnetic beads (Pierce, Thermo #88816) were pre-washed twice with 1.0 mL no-SDS lysis buffer and then incubated with protein lysates for 1 h at RT with gentle shaking (300 rpm). Enriched beads were washed twice with 1 mL no-SDS lysis buffer, once with 1 M KCl, and five times with 100 mM TEAB buffer. Washed beads were resuspended in 50 uL of elution buffer (4 % SDS, 25 mM TEAB, 75 mM NaCl, 20 mM Biotin) and incubated at 99oC for 10 min with gentle shaking (300 rpm). Supernatant was collected. Beads were rinsed with 20 uL of 100 mM TEAB and supernatants were combined. Trace amounts of beads were removed once by magnetization and a tube change.

### 5.11. Proteomics

Eluted peptides from Co-IP were reduced, alkylated, and subject to trypsin digestion on S-Trap Micro’s (Protifi) per manufacture protocol. The resulting peptides were desalted with ZipTips (Sigma Aldrich), dried down, and taken up in 10 μl 97 % H2O, 3 % Acetonitrile, 0.1 % formic acid. A pooled control of all samples was created to monitor instrument variability during LC-MS/MS analysis. 4 μl of each sample (or pooled control) was loaded onto an EASY C18, 1.7 μm 2.1×50cm column at 300 nL/min with an UltiMate™ 3000 RSLCnano HPLC system, eluted over a 120-min gradient and analyzed on Orbitrap Eclipse™. For peptide quantification, the instrument was operated in data dependent acquisition. MS1 spectra were acquired at a resolving power of 240,000. MS2 spectra were acquired in the LTQ with HCD normalized collision energy = 28. Peptides were identified and quantified in Proteome Discoverer (2.5, ThermoFisher). Peptide and protein identification was filtered at an FDR < 0.01. Intensity was used as quantitative metric and normalized to “total peptide amount” in Proteome Discover. Data was exported to excel spreadsheets as “Peptide Groups”.

For proximity label samples, the biotinylated proteins were subject to quantitative mass spectrometry and informatic processing as described above for Co-IP samples, only with peptide intensities exported as peptides-spectral matches.

### 5.12. Data analysis

Saintq (Choi et al., 2011; Teo et al., 2016) is an algorithm to calculate the probability of a protein-protein interaction according to the abundance data from Co-IP-MS data. SAINTq was chosen for its ability to effectively control false positives while identifying true protein-protein interactions in large-scale proteomic datasets. It was utilized here to calculate the posterior probability of a true protein-protein interaction (vs non-specific binding) with an associated false discovery rate using default parameters and supervised model. Each experimental group was compared to no-antibody control individually, then the probability scores were averaged across replicates. For a given protein, if a minimum of 3 peptides were detected across all replicates, it was considered identified in MS. Data imputation for missing values and normalization was performed by Saintq. The SAINTq Bayesian false discovery rate (BFDR) threshold was set at ≤0.05 to limit false positive identifications.

### 5.13. Functional annotation analysis

To determine the biological meanings of our dataset, it was subjected to functional annotation analysis. Proteins that exclusively interacted with WT MAP2 (i.e. demonstrated a loss of binding in S1782E−/+ mice) were entered into DAVID (https://david.ncifcrf.gov/tools.jsp). All detected mouse auditory cortex proteins (Table S5) were set as the background to run a functional annotation analysis using the default settings. An identical analysis was conducted for the 544 WT MAP2 interactors present in common in the Co-IP and proximity label datasets.

### 5.14. Gene set association with schizophrenia

To determine whether proteins identified as MAP2^WT^ or MAP2^S1782E^ interactors were associated with schizophrenia, we performed a gene-set analysis using the Functional Mapping and Annotation of GWAS (FUMA) platform (Watanabe et al., 2017). The input gene set consisted of 920 proteins derived from our Co-IP data. FUMA cross-referenced them with published genome-wide association studies (GWAS) for schizophrenia. Specifically, FUMA utilized the NHGRI-EBI GWAS Catalog as the reference database to identify associations (MacArthur et al., 2017).

## Supplementary Material

Supplement2

Supplement3

Supplement1

Supplement4

Supplement5

Supplement6

Supplementary data to this article can be found online at https://doi.org/10.1016/j.nbd.2024.106731.

## Figures and Tables

**Table 1:** Plasmids used in the Experiments.

Plasmid	Function	Manufacturer	Catalog number

pCMV6-KIF5C	Expressing human KIF5C-Myc-DDK protein (Uniprot: O60282)	Origene	RC218796
pCMV6-KLHL8	Expressing human KLHL8-Myc-DDK protein (Uniprot: Q9P2G9)	Origene	RC207372
pcDNA3.1-PPM1L	Expressing human PPM1L-Myc protein (Uniprot: Q5SGD2)	Genscript	OHu02091
IRES-MAP2B-GFP	Expressing human wild type DDK-MAP2B (Uniprot: P11137–1)	GeneCopoeia	CS-E2438-M61–01

## Data Availability

Data will be made available on request.
