# Supplementary material for: Deciphering the alteration of MAP2 interactome caused by a schizophrenia-associated phosphorylation
Source: Neurobiol Dis. Author manuscript; Available in PMC 2025 Sep 6. (PMC12413633; doi:10.1016/j.nbd.2024.106731)
Supplement: Supplement1 [file NIHMS2041103-supplement-Supplement1.docx]

**
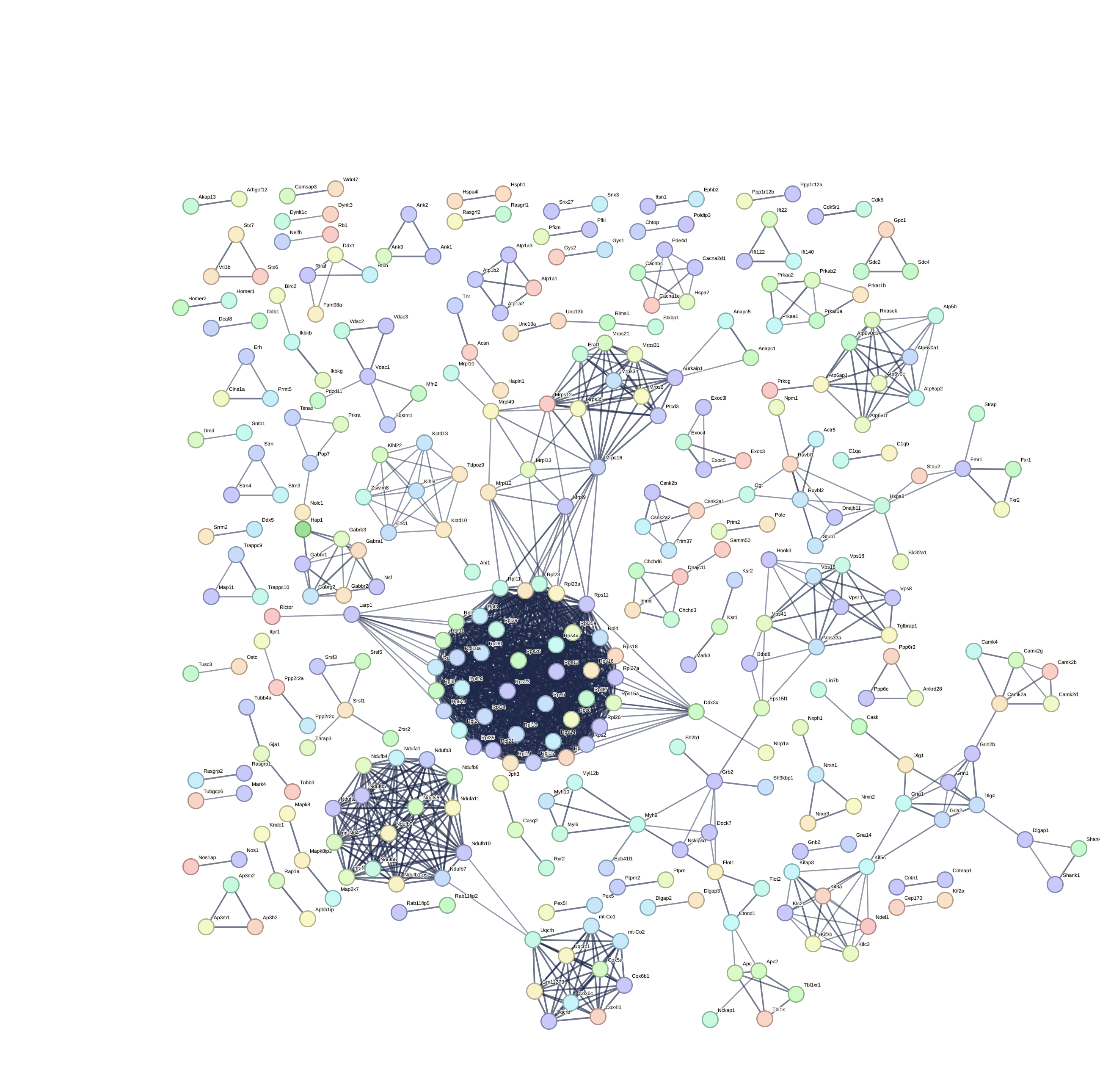
**

**Figure S1 Interaction network of the WT MAP2 interactome in mice.** The network was generated using STRING to visualize the protein-protein interactions within the MAP2 interactome using high confidence (≧ 0.700). Each node represents a protein, and edges indicate that the proteins are part of the same physical complex, based on experimental data and computational predictions. The thickness of the edges indicates the level of confidence. Proteins disconnected to the network were hidden.


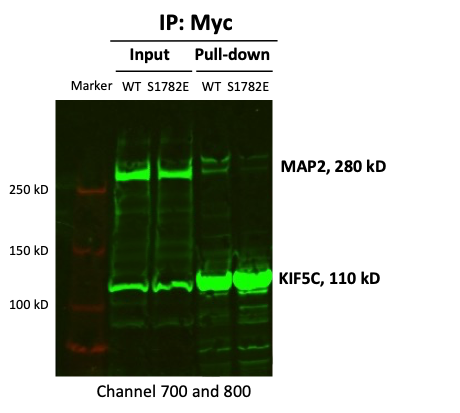


**Figure S2** **Raw image depicting immunoblotting results for Figure 2A.** The image displays a single blot captured at both channel 800 and channel 700 wavelengths, illustrating the fluorescent signal corresponding to the target proteins analyzed.


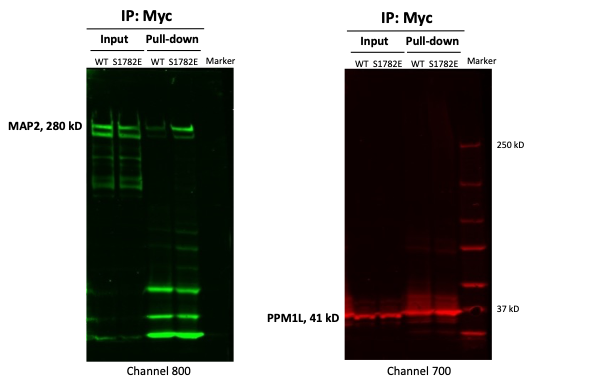


**Figure S3 Raw image depicting immunoblotting results for Figure 2B.** The image displays a single blot captured at both channel 800 and channel 700 wavelengths, illustrating the fluorescent signal corresponding to the target proteins analyzed.

**Figure S4 Raw image depicting immunoblotting results for Figure 2C.** The image displays a single blot captured at both channel 800 and channel 700 wavelengths, illustrating the fluorescent signal corresponding to the target proteins analyzed.


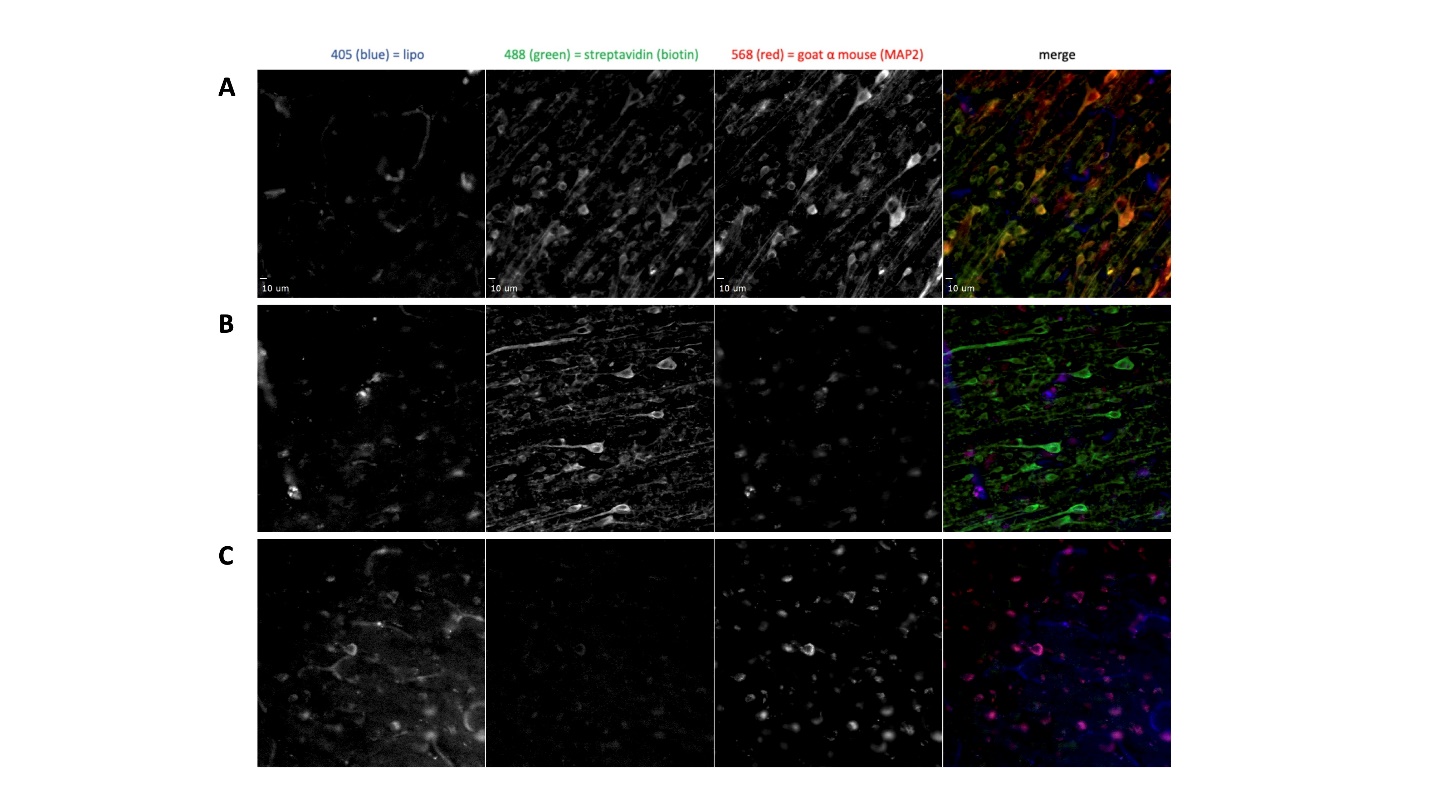


**Figure S5 Confocal images demonstrate the specificity of MAP2 immunolabeling in human brain section.** Tissue sections were stained with MAP2 primary antibodies and secondary antibodies conjugated to Alexa 568 (red) for MAP2, Alexa 488 (green) for streptavidin-biotin detection. A: Sections treated with MAP2 antibodies and Alexa 568 goat anti-mouse secondary antibodies show robust MAP2 labeling (red) with clear neuronal labeling patterns. Streptavidin (green) is also present, with merged images showing overlapping channels. B: Sections treated with MAP2 antibodies but not Alexa 568 secondary antibody show no MAP2 labeling. Streptavidin is present. C: Sections treated with no MAP2 antibody.
